# Correction: Air pollution in the places of *Betula pendula* growth and development changes the physicochemical properties and the main allergen content of its pollen

**DOI:** 10.1371/journal.pone.0352100

**Published:** 2026-06-17

**Authors:** The PLOS One Staff

Fig 1 is incorrect. Please see the correct Fig 1 here.

In the Study sites subsection of Materials and methods, there is an error in the third sentence of the third paragraph. The correct sentence is: In this location, during winter and spring periods, the level of air pollution caused by NOx was up to three times higher than the values recorded in other, also important communication routes of the city of Krakow (BA, RU), and approximately from six to seven times higher compared to the results recorded in TR (an urban agglomeration about three times less-populated than Kraków) (S1 Fig).

In the Chlorophyll *a* fluorescence subsection of Results and discussion, there is an error in the first sentence of the second paragraph. The correct sentence is: Comparison of the maximum photochemical efficiency of PSII of *B. pendula*, Fv/Fm (Fig 1A) shows a similar efficiency of the light-dependent processes of photosynthesis in plants growing on all sites.

In the Discrimination ^13^C subsection of the Results and discussion, there is an error in the first sentence. The correct sentence is: it is assumed that δ^13^C values for C_3_ plants range from −20 to −35‰ (on average −27.5‰) [11, 57] In all localities, δ^13^C values oscillated near the 28.76 ± 1.24‰, which indicates that these plants were in very good physiological conditions and were able to bind CO_2_ directly, with the predominant involvement of the ribulose-1,5-bisphosphate carboxylase oxygenase enzyme (RuBisCO), Fig 1C.

There are errors in the Author Contributions. The correct contributions are:

**Conceptualization:** Dorota Myszkowska, Andrzej Skoczowski.

**Formal analysis:** Iwona Stawoska, Dorota Myszkowska, Jakub Oliwa, Aleksandra Wesełucha-Birczyńska, Monika Ziemianin.

**Funding acquisition:** Iwona Stawoska, Dorota Myszkowska, Monika Ziemianin.

**Investigation:** Iwona Stawoska, Dorota Myszkowska, Jakub Oliwa, Andrzej Skoczowski, Aleksandra Wesełucha-Birczyńska, Diana Saja-Garbarz, Monika Ziemianin.

**Methodology:** Iwona Stawoska, Dorota Myszkowska, Andrzej Skoczowski, Monika Ziemianin.

**Project administration:** Iwona Stawoska, Dorota Myszkowska, Monika Ziemianin.

**Supervision:** Dorota Myszkowska, Andrzej Skoczowski.

**Visualization:** Iwona Stawoska, Dorota Myszkowska, Jakub Oliwa.

**Writing – original draft:** Iwona Stawoska.

**Writing – review & editing:** Iwona Stawoska, Dorota Myszkowska, Jakub Oliwa, Andrzej Skoczowski, Aleksandra Wesełucha-Birczyńska.

S1 Fig is omitted from the list of Supporting Information. It can be viewed below.

The publisher apologizes for the errors.

**Fig 1:**
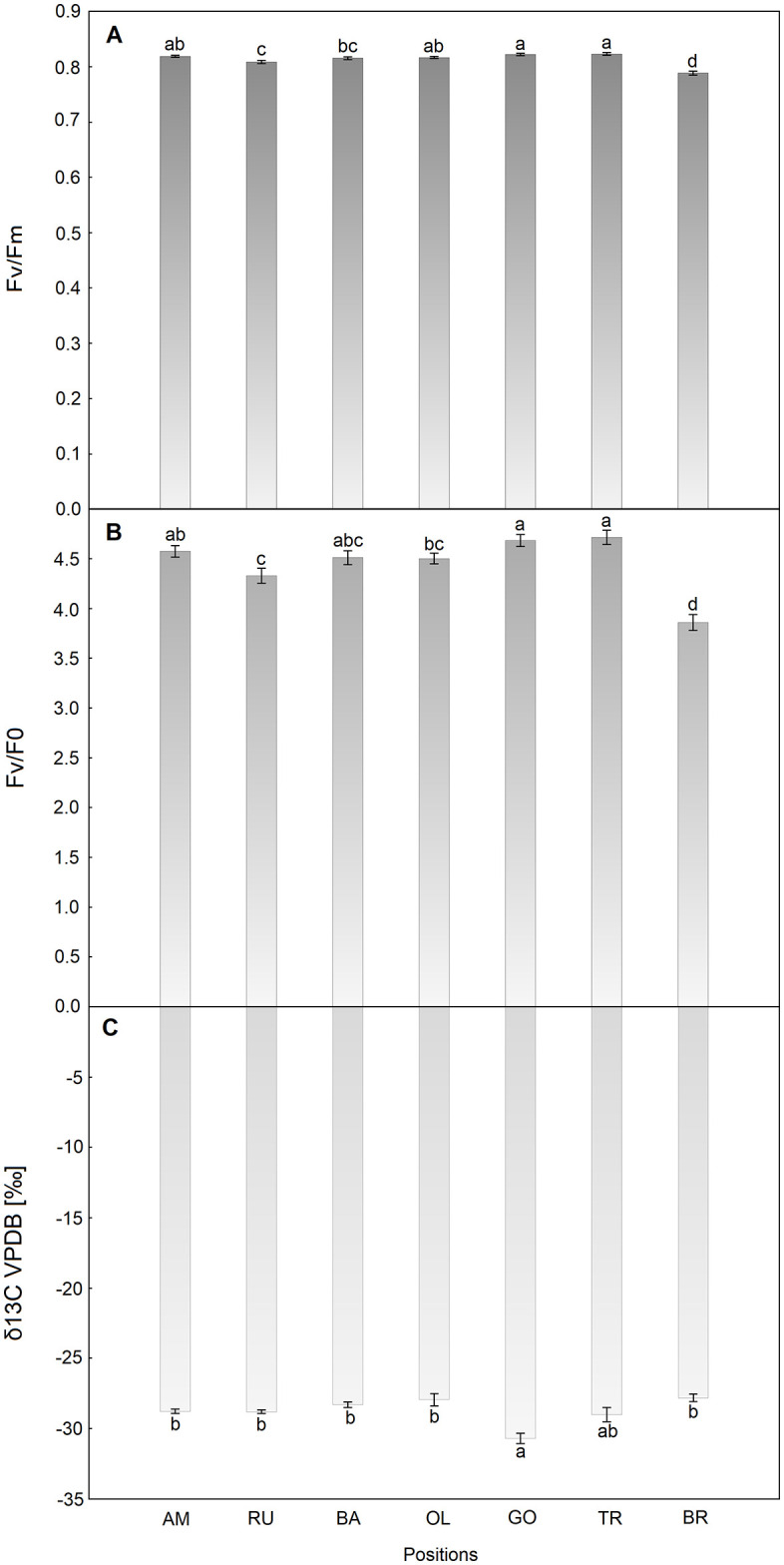
Influence of the growth position in Malopolska region on the values of the parameters characterizing the physiological state of *Betula pendula* plants. **A**. Changes in the maximum quantum yield of photosystem II (Fv/Fm) in leaves. **B**. Differences in the efficiency of the water splitting complex on the photosystem II donor side (Fv/F_0_). **C**. Discrimination of ^13^C carbon isotope in inflorescences of *B*. *pendula* plants. Average values (±SD) marked with the same letters do not differ significantly according to Duncan’s test, p ≤ 0,05. RU–Ruczaj, BA–Batowice, AM–al. Mickiewicza, OL–Olkusz, TR–Trzebinia, GO- Gorlice, BR–Brunary.

## Supporting information

S1 Fig The average (±SD) concentrations of selected pollutants. Daily data collected at six monitoring stations at the Malopolska region, Poland in 2019 from January to September. The whole data were collected in 2017–2019, and Figure 1 SM shows typical pollution changes, graphically illustrated for data from 2019 but repeatable in 2017–2019). Only PM10 data were measured in all studied cities.
(JPG)
