# Supplementary figures and images for: Air pollution in the places of Betula pendula growth and development changes the physicochemical properties and the main allergen content of its pollen
Source: PLoS One. 2023 Jan 25;18(1):e0279826. doi: 10.1371/journal.pone.0279826 (PMC9876359; doi:10.1371/journal.pone.0279826)

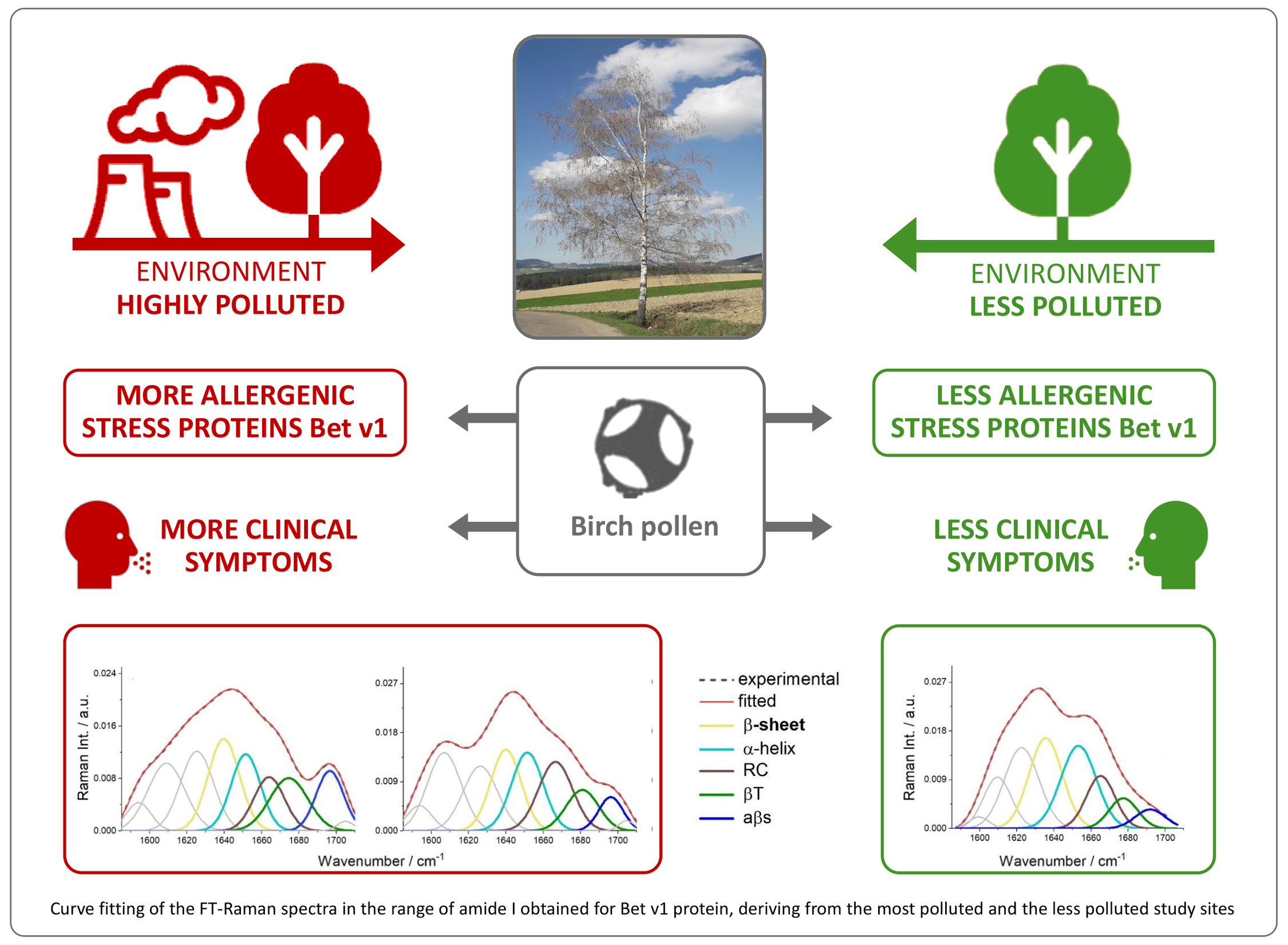

Supplement: S1 Graphical abstract — (TIF) [file pone.0279826.s001.tif]
