# Supplementary material for: Air pollution in the places of Betula pendula growth and development changes the physicochemical properties and the main allergen content of its pollen
Source: PLoS One. 2023 Jan 25;18(1):e0279826. doi: 10.1371/journal.pone.0279826 (PMC9876359; doi:10.1371/journal.pone.0279826)
Supplement: Attachment — Submitted filename: Responce to reviewers.docx_.docx [file pone.0279826.s002.docx]

**Editor Comments**

1. Please ensure that your manuscript meets PLOS ONE's style requirements, including those for file naming.

Authors: The whole manuscript, including graphics was adopted to the PLOS ONE style requirements.

2. The information of no role of the funders in the study should be added to the financial disclosure: “The study was supported by the grant of the National Science Centre, No2016/21/N/NZ8/01369. Grant holder was the corresponding author: Monika Ziemianin”.

Authors: It was indicated in the Cover letter, that the study was supported by the grant of the National Science Centre, No2016/21/N/NZ8/01369. Grant holder was the corresponding author: Monika Ziemianin. The FT-Raman measurements were financed by DNWZ.711.58.2022.PBU statutory research project of Pedagogical University of Krakow, Poland, led by Ph.D. Iwona Stawoska. In both cases, the funders had no role in study design, data collection and analysis, decision to publish, or preparation of the manuscript.

3. We note that you have provided funding information that is not currently declared in your Funding Statement. However, funding information should not appear in the Acknowledgments section or other areas of your manuscript. We will only publish funding information present in the Funding Statement section of the online submission form….

Authors: The information of the funding statement was removed from the text, while within the Cover Letter the appropriate note was included. Please, change the online submission form on my behalf.

4. In your Data Availability statement, you have not specified where the minimal data set underlying the results described in your manuscript can be found. PLOS defines a study's minimal data set as the underlying data used to reach the conclusions drawn in the manuscript and any additional data required to replicate the reported study findings in their entirety. All PLOS journals require that the minimal data set be made fully available. For more information about our data policy, please see http://journals.plos.org/plosone/s/data-availability.

Upon re-submitting your revised manuscript, please upload your study’s minimal underlying data set as either Supporting Information files or to a stable, public repository and include the relevant URLs, DOIs, or accession numbers within your revised cover letter. For a list of acceptable repositories, please see http://journals.plos.org/plosone/s/data-availability#loc-recommended-repositories. Any potentially identifying patient information must be fully anonymized.

Important: If there are ethical or legal restrictions to sharing your data publicly, please explain these restrictions in detail. Please see our guidelines for more information on what we consider unacceptable restrictions to publicly sharing data: http://journals.plos.org/plosone/s/data-availability#loc-unacceptable-data-access-restrictions. Note that it is not acceptable for the authors to be the sole named individuals responsible for ensuring data access.

We will update your Data Availability statement to reflect the information you provide in your cover letter.

Authors: The whole material, including row data, pollen data, electrophoresis pictures, Bet v1 concentrations, FT Raman spectra and all other results measured in frame of the study were stored as project documentation by the National Science Centre. PM10 data were obtained from free open database of the Malopolska Inspectorate for Environmental Protection in Krakow (http://monitoring.krakow.pios.gov.pl). The datasets used and analyzed during the current study are available from the corresponding author upon the request.

5. Please review your reference list to ensure that it is complete and correct. If you have cited papers that have been retracted, please include the rationale for doing so in the manuscript text, or remove these references and replace them with relevant current references. Any changes to the reference list should be mentioned in the rebuttal letter that accompanies your revised manuscript. If you need to cite a retracted article, indicate the article’s retracted status in the References list and also include a citation and full reference for the retraction notice.

Authors: The typos error correction have been made. One position was added (no 47), therefore, positions 47 to 93 have been moved 1 forward.

**Additional comments**

There are several typographical mistake in the text and table. Address then accurately. For instance, minute symbol of table, ml, µl, space between number and degree symbol, etc.

Authors: The whole manuscript was checked for these mistakes and correction was introduced.

**Reviewer Comments to the Author**

Reviewer #1: This paper describes a study about the possible relationship between the physiological conditions of Betula pendula and the air pollution. This is a rather relevant contribution to the better understanding of the problem of increasing allergy of pollen in polluted atmospheres. A particularly important feature of this study is the acquisition of primary experimental data, which is not common. I found only one problem with the analysis of the results. Indeed, because we have a multivariate data set, and besides the univariate analysis that has been presented in the paper, a unsupervised multivariate data analysis should be done, for example principal components analysis, non-linear maps, etc. This would improve the data analysis and interpretation of the results.

Authors: Thank you very much for this suggestion. Indeed, we have a multivariate data set and we considered the possibility of performing the principal component analysis to assess the impact of the environmental pollution parameters on the physiological conditions of plants. However, the data obtained from the measuring stations contain a different number of parameters, which we noted in the manuscript text. For this reason, we don't have complete data to perform the analysis. Moreover, the parameters Fv/Fm and Fv/Fo are interdependent, so introducing them to the analysis seems to be pointless. Taking into account the slight physiological differentiation between the examined objects from different sites, we decided that such an analysis would not bring any breakthrough information to the manuscript.

Reviewer #2: The manuscript "Possible relationship between the physiological conditions of Betula pendula and physicochemical properties of its pollen at selected sites in Małopolska regions" showed
that air pollution has a substantial impact on qualitative and quantitative results regarding chemical compounds of pollen and on the secondary structure of Bet v1. This finding is definitely going to add new knowledge in the field of pollen allergy. However, to make manuscript more effective I have some concerns that need to be addressed.

Authors: Dear Reviewer, thank you very much for your kind opinion. The detailed answers are presented below.

I. There are few typos errors throughout the manuscript that need to be revised.

Authors: The indicated errors were corrected.

II. Abstract is not structured, it is better to make it structured as per the policy of journal.

Authors: Abstract was structured, as suggested.

III. Figure quality and legends need to be revised for clarity.

Authors: The quality of Figures 1SM, 4 and 5 has been improved. The tiny typos were corrected.

IV. The word literature may be replaced by references as per journal guidelines.

Authors: Was done.

V. References are not uniform revise it and make uniform.

Authors: The references list was corrected. Journal title abbreviations were used according to Medline, <https://www.ncbi.nlm.nih.gov/nlmcatalog/journals>

Overall, the manuscript is presented comprehensively and with the suggested minor changes, I recommend acceptance.

Reviewer #3:

Authors: Dear Reviewer, thank you very much for your kind opinion. The detailed answers are presented below.

A more clear title should be inserted.

Authors: The title was rearranged to be more precise.

The abstract should be structured in Introduction, methods, Results, Conclusion.

Authors: Abstract was structured, as suggested.

The aim and novelty character of paper should be better marked.

Authors: The aim of the study was rearranged.

A graphical scheme of study approach should be inserted.
Authors: Graphical abstract was added.

Ftir raman spectroscopy results should be better discussed.

Authors: During the research, we did not perform FTIR measurements, but only FTR, so we are not able to present comparative analysis, because, if we understood the suggestion correctly, this was what the Reviewer meant. In the FTR technique, the 1064nm laser excitation was used (in the infrared range). The lowering of the energy of the laser radiation allows for the reduction of the fluorescence signal and as a consequence improves the quality of the spectrum.

These techniques (FTIR and FTR) are complementary so we haven't seen the advisability of doing both of them. The Raman spectroscopy method, using the Raman effect, successfully competes with FTIR spectroscopy, as it has a wider application and does not require drying the samples. Therefore, it would be impossible to perform FTIR measurements on liquid (aqueous) samples of Bet v1 protein due to the limitation of the IR method.

According to the Reviewer’s suggestion, we have added an additional explanation to the manuscript.

Limits, advantages and practical applications should be better highlighted in Conclusion.

Authors: A short note on practical application of the presented results was added.

6. PLOS authors have the option to publish the peer review history of their article ([what does this mean?](https://journals.plos.org/plosone/s/editorial-and-peer-review-process#loc-peer-review-history)). If published, this will include your full peer review and any attached files.
Authors: In our opinion, it is not necessary to publish the peer history of our article
If you choose “no”, your identity will remain anonymous but your review may still be made public.
