# Supplementary figures and images for: Correction: Air pollution in the places of Betula pendula growth and development changes the physicochemical properties and the main allergen content of its pollen
Source: PLoS One. 2026 Jun 17;21(6):e0352100. doi: 10.1371/journal.pone.0352100 (PMC13274810; doi:10.1371/journal.pone.0352100)

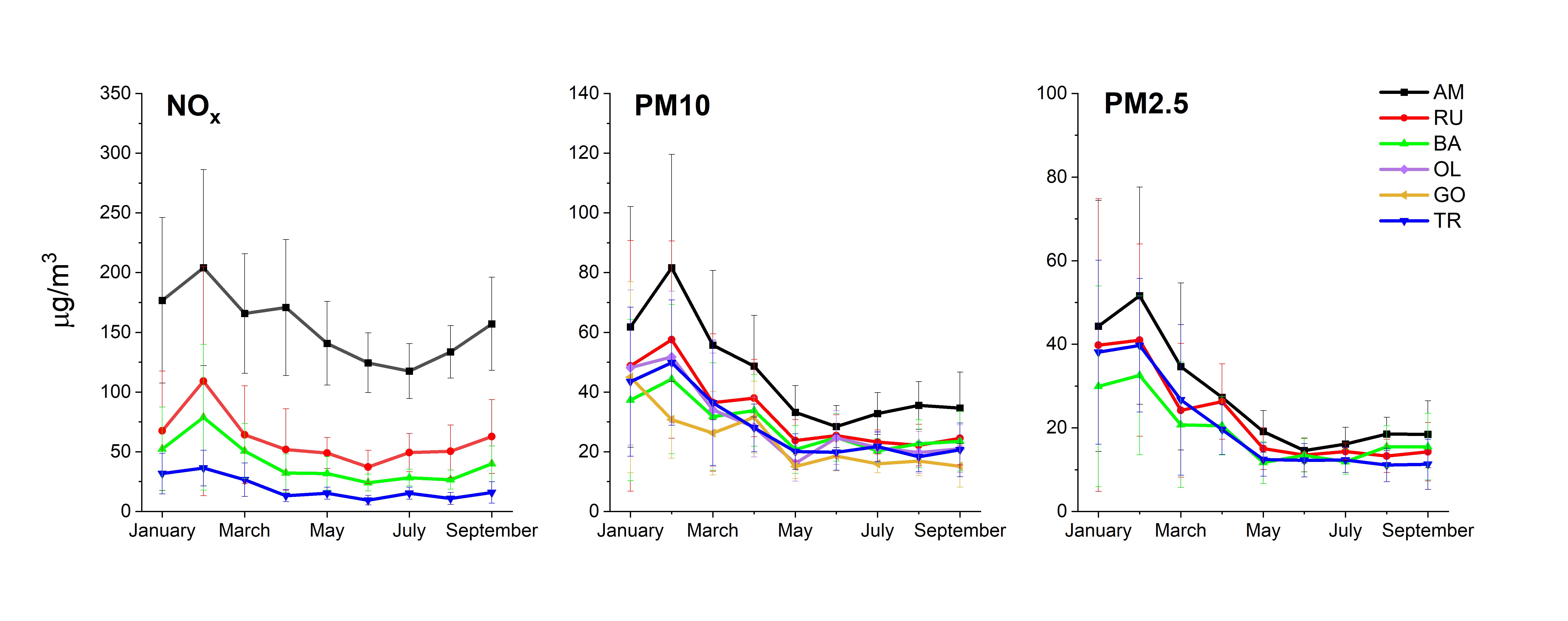

Supplement: S1 Fig — Daily data collected at six monitoring stations at the Malopolska region, Poland in 2019 from January to September. The whole data were collected in 2017–2019, and Figure 1 SM shows typical pollution changes, graphically illustrated for data from 2019 but repeatable in 2017–2019). Only PM10 data were measured in all studied cities. (JPG) [file pone.0352100.s001.jpg]
